# Engineering the common cold to be a live-attenuated SARS-CoV-2 vaccine

**DOI:** 10.3389/fimmu.2022.871463

**Published:** 2022-09-08

**Authors:** Laura M. Kasman

**Affiliations:** Department of Microbiology and Immunology, Medical University of South Carolina, Charleston, SC, United States

**Keywords:** live vaccine, SARS-CoV2, replication competent, human, rhinovirus, intranasal vaccination, attenuated

## Abstract

According to the American Centers for Disease Control and Prevention, people in all age groups catch two or more “colds” per year, at least half of which are caused by human rhinoviruses. Despite decades of effort, there are no vaccines or drugs against rhinovirus infections and even social distancing measures that were effective in reducing the spread of the pandemic coronavirus, SARS-CoV-2, did not reduce the rate of rhinovirus detection. Fortunately, most rhinovirus strains are naturally attenuated in that they are not associated with serious illness, hospitalization or mortality. Instead, rhinoviruses are one of the most frequent viruses found in nasal swabs of asymptomatic, healthy people. Since rhinovirus infections cannot be avoided, a rational approach would be to engineer them for the benefit of their human hosts. Rhinovirus infections naturally induce robust mucosal and serum immune responses to all virus-expressed proteins. Several replication-competent, human rhinovirus vaccine vectors able to express protective antigens for other pathogens have already been designed and tested in animal models. With this strategy, the inevitable common cold would be able to induce immunity not just to a specific rhinovirus serotype but to other more pathogenic respiratory viruses as well. This article reviews existing rhinovirus vaccine vector technology and describes the characteristics that make live-attenuated rhinoviruses attractive vaccine candidates for SARS-CoV-2 and other pathogenic respiratory viruses in the future.

## Introduction

The prolonged nature of the SARS-CoV-2 pandemic in an era with unprecedented capacity for data collection and analysis resulted in a remarkable pace of discovery, including the production, testing and worldwide distribution of multiple effective vaccines. However, all of the SARS-CoV-2 vaccines to date require one or more injections, cold storage, and a sophisticated distribution network of trained personnel for administration (1). Human rhinoviruses (HRV), even more than coronaviruses, spread from person to person despite diligent efforts at prevention. During the 2019-2021 SARS-CoV-2 pandemic, social distancing and masking protocols to reduce coronavirus transmission resulted in significantly lower numbers of respiratory syncytial virus and influenza virus infections. This was not the case for HRV (2–4). Rhinovirus infections are currently an inevitable part of life for humans of all ages, with an expected one to two rhinovirus infections per year over an average lifespan (5). Once recovered, immunity to that specific rhinovirus serotype will be strong, but with over 100 known serotypes, future symptomatic rhinovirus infections are inevitable (6). While discouraging from the point of view of preventing colds, seen another way it is an opportunity. Non-pathogenic rhinovirus vectors, with so many serotypes to choose from, relatively benign symptoms, and characteristics that favor immunization *via* nasal spray or eye drops, may provide a means to fight fire with fire in the next pandemic. The technology already exists to make it possible for the unavoidable annual rhinovirus infection to induce immunity not just to that specific rhinovirus serotype but to other more pathogenic respiratory viruses as well. The following sections provide a summary of rhinovirus biology with regard to the safety and feasibility of engineering them to be a live-attenuated vaccine against SARS-CoV-2 and other pathogenic respiratory viruses.

## Rhinovirus epidemiology and biology

In non-pandemic years, HRV cause more than half of all symptomatic upper respiratory infections worldwide and year-round (5, 6). The illnesses they cause are estimated to cost billions of dollars in medical visits and missed days of work each year, but they remain extremely rare causes of mortality in any age group, and far less than other respiratory pathogens (5). Over 100 HRV serotypes have been identified, which are divided into three species or clades designated HRV-A, -B and -C by phylogenetic analysis (7).

The genomes of HRVs are tiny and simple, functioning as a single mRNA molecule of approximately 7200 bp that encodes a single open reading frame. All 11 proteins that the virus requires for propagation are encoded within this single sequence, including two proteases that become active as the nascent protein folds. These proteases, designated 2A and 3C, cleave the polypeptide in a carefully conserved progression, ultimately resulting in assembly of progeny virions (5, 7). HRV virions are unenveloped icosahedral capsids assembled from 60 copies each of four proteins, VP1-4. VP1 encodes the attachment protein. Unlike enveloped respiratory viruses which usually encode attachment sites that protrude from the virion surface, the HRV attachment point to the host cell is located within an indentation on the capsid surface. Each HRV species utilizes one of three different host cell receptors for attachment and entry: ICAM-1, low-density lipoprotein receptor (LDLR) or cadherin-related family member 3 (CDHR3) (7–9). ICAM-1 is the major receptor, utilized by 88 serotypes (5, 7). All CDHR3 binding strains are HRV-C, while all LDLR-binding strains are HRV-A, and ICAM-1-binding strains are both A and B (7).

Rhinoviruses are genetically stable, increasing their safety as a vaccine vector. A prototype collection of 99 serotypes collected in the 1960’s and 70’s from clinical specimens has remained the canonical serotype set for HRV species A and B to this day (5). It was sequenced in 2009 and all types are still in circulation (7). The third species, HRV-C, was discovered by molecular techniques in 2006 and likewise appears to be genetically stable (9). Rhinovirus recombination between serotypes is much less common than in other enterovirus types (10). Based on genomic sequence analysis, recombination events between HRV have occurred in the distant past, but they are relatively rare (7). This genetic and phenotypic stability may be a result of the fact that human rhinoviruses have no known non-human reservoirs, unlike influenza and coronaviruses. Regardless of the mechanism, new rhinovirus antigenic variants are extremely rare.

## Rhinovirus transmission

HRV are transmitted by respiratory droplets, but more often they are transmitted by virus particles on the hands coming into contact with the nasal or conjunctival mucosa (5). A curious finding from the COVID-19 pandemic is that HRV incidence was not significantly reduced by masking and social distancing measures that reduced other respiratory viral infections (2–4) The reasons for this are not yet determined, but infections with enveloped respiratory viruses were reduced more than non-enveloped, such as rhinoviruses and coxsackieviruses (2). Therefore a likely explanation for continued HRV transmission despite social distancing is its greater stability on touch surfaces and consequently greater transmission of HRV *via* fomites and hand-to-face contact. Taken together, rhinoviruses appear to be ideal for immunization *via* nasal spray or eye drops.

## Immunity to rhinovirus

Immune responses to HRV have been studied extensively and recently reviewed (6). Experimental HRV infections in human volunteers elicit robust innate, humoral and cell-mediated immune responses (5). The virus activates multiple innate pattern recognition receptors in the upper respiratory tract, resulting in type I and type III interferon expression, as well as inflammatory cytokines and chemokines (6). HRV-specific serum (IgG) and mucosal (IgA) antibodies are detected within 1-2 weeks, which is usually after symptoms have resolved (5). Antibodies do provide protection against future infections with the same serotype HRV for at least a year and probably longer, by blocking attachment of virus particles to the mucosal epithelium (6). Both cytotoxic and helper T-cell immunity is induced. T-cell responses are directed against both structural and non-structural viral proteins and are more cross-reactive between serotypes than antibodies (11, 12). In individuals who clear the virus efficiently, HRV-specific CD4+ T-cells are mostly of the TH_1_ type, whereas a TH_2_ phenotype dominates in most individuals with asthma (6). There is evidence that HRV-C is much more likely to induce TH_2_ responses than HRV A and B (8), so HRV-C strains should be avoided as vaccine vectors.

The long-lasting serotype specific immunity to HRV means that any HRV serotype chosen as a vaccine vector may be less effective in some fraction of the population that has prior exposure to it. Developers of the first rhinovirus vaccine vectors took this into account and when possible chose rare serotypes (13). The large number of HRV serotypes available means that this is less of a barrier for HRV than for other virus vectored vaccines

## Rhinovirus engineering and production potential

Utilization of recombinant, replication competent human rhinovirus vaccines was investigated as early as 1994 due to the ability of wild type rhinovirus to induce robust mucosal and serum immune responses, the safety of the infection, and the ease of nasal inoculation to achieve immunization (14). Obstacles that prevented HRV from being widely considered as a vaccine vector are its limited capacity for inserted sequences, lack of available small animal models, and perceived pre-existing immunity in the general population (13). However, two approaches to engineering human rhinoviruses as vaccine vectors against human immunodeficiency virus (HIV) provided evidence of efficacy in preclinical studies (**Table 1**).

**Table 1:** Rhinovirus vaccine vectors.

Reference	Vaccine Antigen(s)	Antigen expression type and location	Replication competent	*In vivo* trials
Arnold GF, et al. (1994)	Fragments of poliovirus 3 Sabin VP1, poliovirus 3 Sabin VP2, influenza hemagglutinin, HIV virus gp120, HIV gp41	Fusion protein with vaccine antigen inserted between Ala-159 and Asp-160 of the surface loop connecting β strands E and F of the VP2 coat protein of HRV14	3 out of 12 strains (influenza antigens only)	Intradermal inoculation of guinea pigs (an HRV non-permissive species) failed to induce neutralizing antibodies
Resnick DA, et al. (1995)	Library of the 7 amino acid consensus sequence of HIV-1 gp120 V3 loop flanked by 0-2 random amino acids	Fusion protein with vaccine antigen inserted between Ala- 159 and Glu-161 of the surface loop connecting β strands E and F of the VP2 coat protein of HRV14	Yes	Guinea pigs (an HRV non-permissive species) intradermally inoculated with selected virus clones induced antibodies that neutralized HIV *in vitro*
A.D. Smith, et al. (1998)	HIV-1 gp120 V3 loop	Same as Arnold GF, 1994	Yes	Guinea pigs (an HRV non-permissive species) intradermally inoculated with purified chimeric rhinoviruses induced neutralizing antibodies against the target epitope
Arnold GF, et al. (2009)	ELDKWA epitope of the membrane-proximal external region of HIV-1 gp41	Same as Arnold GF, 1994	Yes	Subcutaneous injection of guinea pigs (an HRV non-permissive species) with or without peptide boosters induced neutralizing antibodies against the target epitope
Tomusange K, et al. (2015), Tomusange K, et al. (2016)	HIV Gag fragments and complete Tat protein	P1/P2 junction of the HRV-A1 genome, flanked by viral protease P2A cleavage sequences Vaccine antigen is released *via* viral protease cleavage.	Yes	Intranasal immunization of mice (non-permissive species) with rHRV or WT HRV was followed by intradermal injection with a DNA vaccine in the EcoHIV challenge model. Robust CD8+ T-cells were induced.

The earlier approach expressed potential vaccine epitopes as a fusion with a known immunogenic loop of amino acids exposed on HRV capsid protein VP2. To allow the epitope more freedom to take on its natural conformation, short random amino acid linkers were added, flanking the epitope. Attempts to express large antigens were not successful (14), but rhinoviruses expressing short (12 amino acid) epitopes of HIV are replication competent in HeLa cells and are neutralized by anti-HIV monoclonal antibodies specific for their vaccine epitopes (15, 16). Furthermore, purified virions expressing the HIV-VP2 fusion were used to immunize guinea pigs intradermally and shown to induce neutralizing antibodies against the target epitope (15, 16). Guinea pigs are not permissive for HRV replication, and the intradermal inoculation route did not take advantage of the rhinovirus capacity for mucosal immunization.

In 2015, Tomusange et al. (13) developed a very different rhinovirus vaccine vector by making use of the viral protease P2A. Sequences encoding antigens of interest are inserted at the P1/P2 junction of the genome flanked by sequence encoding P2A cleavage sites (NTITTAG/PSDLY). Unwanted homologous recombination between the duplicate cleavage site sequences is prevented by introduction of strategic silent mutations. Insertion sequences of up to 500 bp result in viruses that are replication competent in Hela and human bronchial epithelial cells and genetically stable (13, 17). During replication, the antigenic peptide is cleaved free from the viral polyprotein and can be detected (17). Intranasal inoculation of P2A-based HRV vectors in a mouse model induced epitope-specific antibodies in blood and mucosal fluids, and strong CD8+ T-cell responses in lymphoid tissues (18). While this animal model was also replication incompetent for HRV, it provides proof of concept for rhinovirus vaccine vectors as mucosal vaccines.

In both existing rhinovirus vaccine vector approaches, the ~7200 bp HRV RNA genome is converted to a cDNA and inserted into a bacterial plasmid cloning vector for addition of sequences encoding vaccine antigens (13–15). Full length transcripts are transcribed in a cell-free reaction and transfected into Hela or other existing human cell lines to produce infectious virus. Once infectious recombinant HRV is produced, it can directly infect permissive host cells for large scale vaccine production. The thermostability of HRV vaccine virions has not been published, but it is likely to be very similar to that of other picornaviruses, such as hepatitis A virus and poliovirus. The live-attenuated Sabin poliovirus vaccine is documented to maintain its viability for six weeks at 22-25°C with no loss in titer (19).

## Epitopes for rhinovirus-based SARS CoV-2 vaccines

Almost all COVID-19 vaccines currently in use or under development present the whole SARS CoV-2 spike protein or the whole virus. The spike protein is 1273 amino acids in length, which requires a genetic sequence far exceeding the 500 bp maximum insert size of a rhinovirus vector (20). However, much shorter peptides are immunogenic and a recent study (21) used multiple bioinformatics databases and tools to derive consensus SARS CoV-2 spike protein epitopes that are predicted to induce both T and B-cell responses in more than 99% of people worldwide. The top four consensus B- and T-cell epitopes are 18, 30, 35, and 39 amino acid residues in length, capable of being encoded with 117 bp or less, well within the capacity of the P1/P2 junction vector. An alternative strategy which assumes no prior knowledge of immunogenicity was demonstrated successfully by Resnick et al. in 1995 (22). In this strategy, a library of VP2-fusion rhinoviruses is made with random inserts of 7-11 amino acids. The library is then selected using antibodies known to strongly neutralize the target pathogen, such as SARS CoV-2, and the viruses with highest affinity are cloned and produced as vaccine strains. Interestingly, when this was done for the HIV V3 loop antigen, the rhinoviruses with highest affinity had amino acid inserts that did not match the sequence of any V3 loop epitopes (22).

## Rhinovirus morbidity and mortality

Rhinovirus infections are self-limiting in the vast majority of cases. However, asthma and COPD exacerbations are strongly associated with both naturally occurring and experimental HRV infections (8, 9, 23, 24). People with asthma have different immune responses to HRV, including innate, TH_1_ and Treg responses, which suggests that an immunogenetic predisposition plays a role (23, 24). In addition, individuals who express a variant of CDHR3, the receptor for HRV-C, are more susceptible to severe HRV-C infections in childhood and developing asthma as a result (9). Because of the adverse outcomes in people with asthma or COPD, multiple capsid binding antivirals and a protease inhibitor specific for HRV protease 3C have been evaluated in human clinical trials with both natural and experimental rhinovirus infection methodologies. A few of these agents reduced viral loads and length of virus shedding, but none significantly reduced symptoms and so were not developed further. There are currently no HRV therapies approved for clinical use, and development of an anti-HRV vaccine is considered impractical due to the large number of serotypes identified (5, 6).

Whether specific HRV strains have different morbidity profiles is controversial, with some reports finding that HRV-C is more severe and others not. A few rhinovirus outbreaks in neonatal ICUs and long-term care facilities have been reported in which pneumonia, and rarely deaths, have occurred, but each outbreak was associated with a different HRV strain (5). In addition, a study of nasal washes from children hospitalized with community acquired pneumonia compared with healthy controls found that rhinoviruses were actually detected more frequently in the healthy children, suggesting that HRV detected by multiplex PCR assays may actually represent asymptomatic shedding rather than the pathogen (25). The fact that rhinovirus transmission was not reduced despite implementation of unprecedented public health measures during the COVID-19 pandemic indicates that a live-attenuated rhinovirus vaccine would not increase rhinovirus morbidity or mortality even if shed into the environment by vaccinated individuals.

Co-infection with two or more respiratory viruses at the same time is important to consider when contemplating a replication competent vaccine. Co-infections are well documented and relatively common in respiratory infections, with 4-19% reported in a recent meta-analysis (26), although they are not necessarily associated with more severe disease. Experimental rhinovirus co-infection inhibits SARS-CoV-2 replication in human bronchial epithelial cells *in vitro* by triggering the interferon-mediated antiviral innate immune response (27). HRV are strong inducers of type I interferons, which in turn initiate the innate antiviral response in nearby, uninfected cells. Cells in the antiviral state express latent kinases and RNAses that when activated by viral pathogen-associated molecular patterns induce apoptosis, aborting virus replication in that host cell (6). Natural rhinovirus/SARS-CoV-2 co-infections have not been able to verify this protective effect due to a low incidence of rhinovirus co-infections and high rate of co-morbidities in patient populations studied, but a meta-analysis also found no adverse effects of rhinovirus co-infection in those with COVID-19 (26, 28).

## Conclusion

Live-attenuated vaccines provide longer-lasting, more robust immunity to the diseases they target. The rotavirus, measles, mumps, rubella and varicella vaccines currently approved for children in the United States are replication competent, live-attenuated viruses that were derived by artificial selection in laboratories. Utilization of a naturally attenuated virus as a human vaccine was recently reported for a new rotavirus strain (29). The vaccine rotavirus strain was originally obtained from stool of asymptomatic children, propagated in culture and safely and effectively used to orally inoculate newborns in a Phase II clinical trial (29). A similar opportunity exists for HRV-based vaccines. Most HRV are naturally attenuated viruses, causing self-limited illness or no symptoms in infected individuals. They are considered so safe that there are currently more than 12 approved clinical trials in which healthy and asthmatic subjects are experimentally infected with tissue culture derived HRV (30).

In addition to their familiarity and safety, rhinoviruses have many other advantages as potential vaccine vectors. They are stable at room temperature for weeks, reducing the need for refrigeration. Natural and experimental rhinovirus infection *via* inoculation of the conjunctiva or nasal mucosal surface, induces a protective, specific, long-lasting mucosal immune response. This means that they are ideal for immunization *via* nasal spray or eye drops, eliminating the need for sterile needles and administration by licensed healthcare providers. They have a simple genome consisting of a single open reading frame, and are easy to grow in culture. Unlike influenza and coronaviruses, rhinoviruses have no non-human reservoirs and new variants are extremely rare. HRV activation of innate immune responses likely inhibits replication of other respiratory viruses, protecting their host. Finally, as with any live-attenuated vaccine, shedding of a rhinovirus vaccine virus by immunized individuals has the potential to accelerate population immunity against SARS-CoV-2 and other virulent respiratory viruses, avoiding future prolonged social and economic disruptions like those resulting from the COVID-19 pandemic. What is lost with a vaccine that can immunize close contacts as well as the vaccinee is precise knowledge of who is vaccinated. However, the pace of pandemics is increasing. At this time, with so much knowledge about the present pandemic virus available and resources mobilized, the time is right to try new strategies.

## Author contributions

LK conceived, researched, and wrote the manuscript.

## Funding

Funds for the publication of this article were provided to the author by the Medical University of South Carolina.

## Conflict of interest

The author declares that the research was conducted in the absence of any commercial or financial relationships that could be construed as a potential conflict of interest.

## Publisher’s note

All claims expressed in this article are solely those of the authors and do not necessarily represent those of their affiliated organizations, or those of the publisher, the editors and the reviewers. Any product that may be evaluated in this article, or claim that may be made by its manufacturer, is not guaranteed or endorsed by the publisher.
